# Importance of Melt Flow Direction during Injection Molding on Polymer Heat Sinks’ Cooling Efficiency

**DOI:** 10.3390/polym13081186

**Published:** 2021-04-07

**Authors:** Michal Guzej, Martin Zachar, Jan Kominek, Petr Kotrbacek, Robert Brachna

**Affiliations:** Heat Transfer and Fluid Flow Laboratory, Faculty of Mechanical Engineering, Brno University of Technology (BUT), Technicka 2896, 616 69 Brno, Czech Republic; martin.zachar@vut.cz (M.Z.); jan.kominek@vut.cz (J.K.); petr.kotrbacek@vut.cz (P.K.); robert.brachna@vut.cz (R.B.)

**Keywords:** polymer heat sink, thermal management, thermal conductivity, composites, anisotropy

## Abstract

Polymers with highly conductive fillers could possibly replace standardly used materials, such as aluminum and copper alloys, for passive cooling purposes. The main problem of the composite polymer-based heat sinks is that their high thermal conductivity is uneven. The orientation of this anisotropy is set according to the position of the highly thermally conductive filler. Its orientation is influenced by the melt flow during the polymer heat sink molding process. This article shows that change of the melt flow inside the mold cavity can improve the overall cooling efficiency of a polymer heat sink, which leads to lower temperatures on the heat source used. Two polymer heat sinks of identical geometries were produced. Their high thermal conductivity was given by the use of graphite flakes as the filler. The only difference between the heat sinks was in the position of the fan gate during their production. Different temperatures of the heat source between the two heat sinks were observed for the same measurement conditions. The measurements were conducted at Heatlab, BUT.

## 1. Introduction

Various polymer materials have found their use where, previously, completely different materials were applied. The use of polymers for heat transfer problems was generally unthinkable, but with newly found solutions, plastics have become more appealing in this area as well [1,2,3]. A lot of new research has been done in the area of thermally conductive polymers [4,5,6,7,8], and one of the possible thermal management uses of these polymers could be as heat sinks for electronics cooling. Research has been done in the area of using thermally conductive polymers as heat sinks in recent years. Marchetto et al. [9] reviewed previous research in this area and the problem of the optimal orientation of a highly thermally conductive filler was studied [10], where using various ways of 3D printing achieved different filler orientation and, therefore, different cooling efficiencies.

If a highly thermally conductive filler is applied, the entire compound also becomes more thermally conductive, according to its orientation. Heat sinks made from highly thermally conductive polymer compounds could be lighter in comparison with standard heat sink materials, and furthermore, they could be produced for a significantly lower cost since lower energy consumption is needed during the production process, which would lead to reduced CO_2_ emissions. Another difference of these composite materials is their high surface emissivity compared to standard aluminum and copper alloys, which leads to an increase in heat transfer via radiation. Furthermore, polymers can easily be molded into a higher variety of geometries, and even small changes can improve their cooling efficiency, as shown in [11,12].

Even with the latest advancements in the increase in polymers’ thermal conductivity, they are still far behind in comparison with standardly used metal materials [13,14]. However, in applications where heat transfer by convection is strictly limited (convective resistance is predominant) due to a small, enclosed volume and lack of additional active cooling, the use of these materials could lead to similar results for a lower price. Areas for possible application include electronics cooling in mobile phones, tablets, and even the automotive industry in LED headlamp cooling systems, as they also create a small, enclosed volume around LEDs which are also heated by the engine.

The goal of mold design is to achieve the required quality of the product and minimize the cost of production. For these composite polymers, it is necessary to take into account the final arrangement of additives in addition to the commonly pursued quality parameters, and to appropriately adjust the melt flow, which means the position of the gate influences the final heat sink’s performance.

This article reports different cooling efficiencies between polymer heat sinks of identical geometry and those produced from the same polymer composite with graphite flakes as the highly thermally conductive filler. The compared heat sinks are produced with a fan gate leading to the base edge of the heat sink, both parallel (the first heat sink) and perpendicular (the second heat sink) to its fins. Furthermore, the article compares the measured heat sinks in two positions: “default” and “upside-down”, which leads to a change in position of the filler orientation in the first heat sink, in terms of direction to gravity and buoyancy. This position change leads to different cooling efficiency even though no visible change can be observed from the outside.

## 2. Materials and Methods

### 2.1. Geometry of Tested Heat Sinks

**Figure 1:**
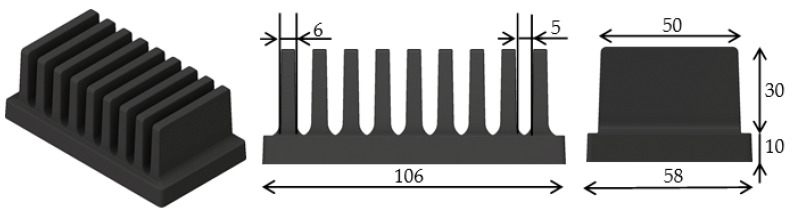
Measured heat sinks’ geometry (dimensions are in mm).

For the heat sinks compared in this article, a commercially available polymer from the company, Avient (formerly PolyOne) which uses the base matrix, Polyamide 66, was used. Its high thermal conductivity was achieved via a highly thermally conductive filler in the form of graphite flakes. The thermal conductivity of this material ranged from 5 to 20 W/m·K, according to the provided data sheet. The importance of the filler orientation in a cross section of a thin plate, such as a heat sink fin, was studied in [15]. However, this article deals with the overall orientation inside the fins, depending on the melt flow during production. Because of its importance for overall cooling efficiency, two polymer heat sinks of the same geometry, but with a different position of the gate during production, were compared. This geometry is shown in Figure 1.The measured heat sinks had a base which was 106 mm long, 58 mm wide, and 10 mm high. Each heat sink had nine 30 mm high and 50 mm long fins that were 6 mm thick.

**Figure 2:**
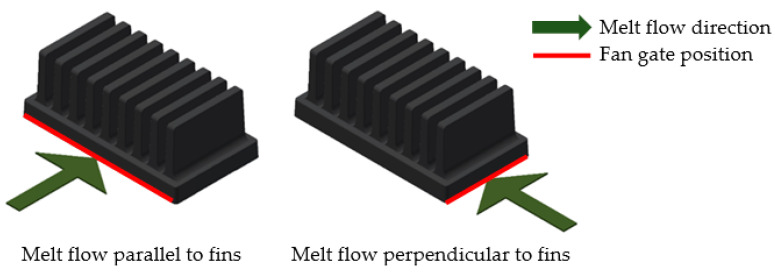
Melt entrance along the edge of the base (red), direction of melt flow depicted by green arrow. (**Left**)—edge of longer base’s side (melt flow parallel to the fins), (**Right**)—edge of shorter base’s side (melt flow perpendicular to the fins).

The only difference between the measured heat sinks was, as mentioned, in the entry of the melt into the mold’s cavity during heat sink production. The first compared heat sink was manufactured with the melt entrance along the edge of the longer heat sink base side (parallel to the heat sink’s fin orientation and buoyancy direction while in use). The second heat sink was produced with the melt leading to the edge of the shorter heat sink base side (perpendicular to the heat sink’s fin orientation and buoyancy direction). The difference in the melt flow entry is shown in Figure 2.

### 2.2. Measurement Description

**Figure 3:**
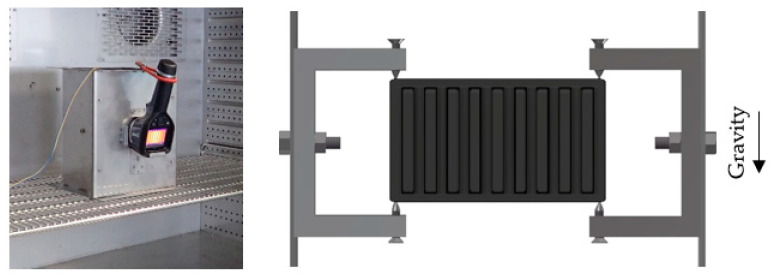
Measurement box inside the thermostatic chamber (**left**), heat sink placement inside the measurement box (**right**).

Heat sinks were placed in a measurement box made for the purpose of heat sink testing, which was put inside a thermostatic chamber, shown in Figure 3. This approach ensured a stable, ambient temperature of 20 °C, without any additional forced convection induced by the chamber’s fans, as well as repeatability of the measurements. Heat sinks were placed in the box in such a manner that a minimal amount of heat from the heat sink would be conducted away via convection. The box’s holders touched the heat sinks only in four points, as is shown in Figure 3. The box’s door had an opening where a thermal camera was placed. For the thermal imagery, a thermographic camera, FLIR E5, was used.

**Figure 4:**
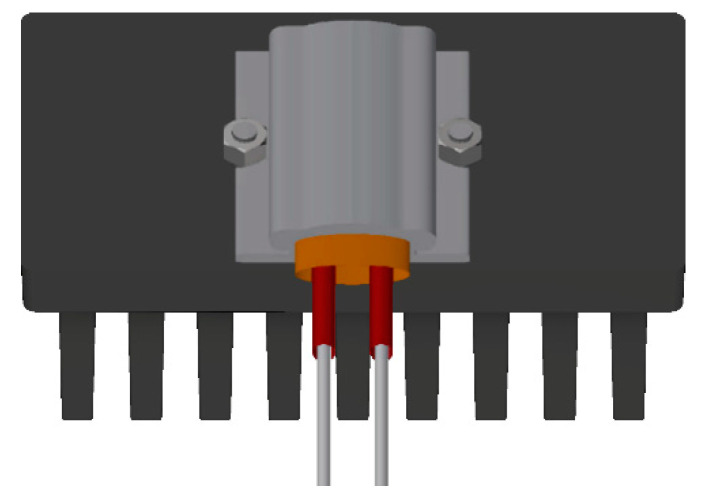
Heater placed in the middle of the heat sink.

A heater was placed on the heat sinks’ bases as shown in Figure 4. The area of the contact surface was 35 mm × 40 mm. An exact amount of electrical power was provided to the heater, which was regulated via a LabView program because the heater’s electrical resistance was increasing with rising temperature. The input power was set to 15 W with a precision of ±0.1 W. A thermal paste with a conductivity of 4.2 W/m·K was applied between the heat sinks’ bases and the heater to minimize the thermal resistance and improve contact between the two. Every measurement was repeated twice, detaching the heater from the base and reattaching it back to ensure the contact between the two was suitable and the same in all conducted measurements, which was checked when measured values were compared between the repeated measurements of the same configurations.

**Figure 5:**
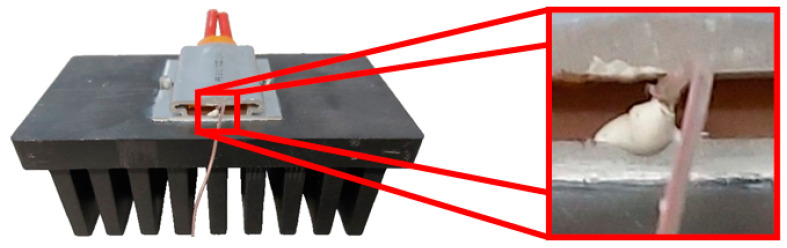
Placement of thermocouple on the heater.

The cooling efficiency results were obtained via a thermocouple of type K placed inside the heater, as seen in Figure 5.

**Figure 6:**
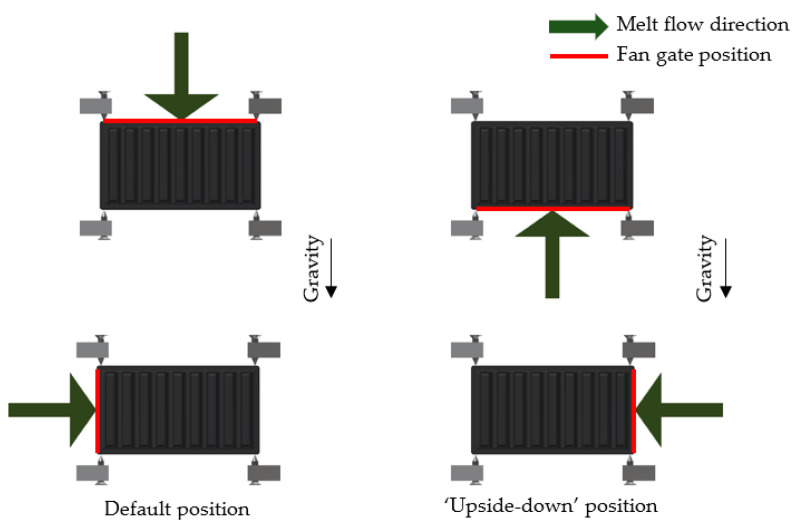
Orientation of the melt entry for both measured heat sinks in two different measurement positions, (**left**)—default position, (**right**)—“upside-down”. Melt entrance along the red edge, melt flow in the direction of the green arrow.

Each heat sink was measured in two positions: “default” and “upside-down”, as is shown in Figure 6. Therefore, the entry of the melt flow would change its position in regard to gravity in the case of the heat sink being produced with the melt entrance along the longer base’s side. In the case of the heat sink being produced with the melt entrance along the shorter base’s side, the entrance only moved from left to right.

## 3. Measurement Uncertainty Analysis

To ensure both temperature measurements by thermocouple and by thermal camera showed correct values, the following steps were executed:

The thermocouples of type K standardly measure with a precision of ±2.2 °C. To achieve a higher precision, the same thermocouple was used in all of the heat source temperature measurements, and this thermocouple was furthermore calibrated for 100 °C to achieve maximum deviation of ±0.2 °C.

**Figure 7:**
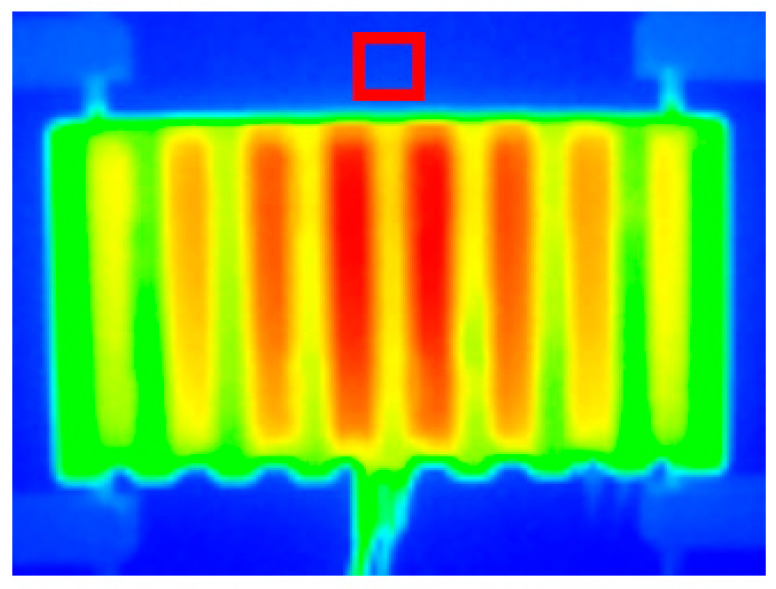
Thermal picture with an indicated area where the thermocouple for the verification of the correct measured temperature via thermal imagery was put.

Thermal images of all the measured heat sinks were taken after a steady state was reached during the measurements. The measurement precision of the thermographic camera, FLIR E5, was ±2 °C with resolution of 120 × 90 pixels. To ensure the measured temperature had higher precision, a different calibrated thermocouple of type K was put into the back wall in a place that was seen in all of the thermal pictures. The wall was dyed with a graphite spray to ensure high and known emissivity. On every thermal picture, an average temperature was measured inside the estimated area where the thermocouple was put, and only the pictures with measurement deviation smaller than ±0.5 °C were considered for the purpose of further heat flow calculations. This area is portrayed in Figure 7.

Precision of the ambient temperature was validated with two resistance thermometers PT100 placed at the bottom of the measurement box that served as an inlet once the air flow was created by the natural convection. The resistance thermometers measured with a precision of ±0.2 °C for the ambient temperature, 20 °C.

## 4. Results

### 4.1. Temperature Comparison

Both compared heat sinks were measured with 15 W of thermal input power from the heater in two positions. The ambient temperature was maintained at 20 °C. The temperatures of the heater are shown in Table 1.

The heat sink with the melt flow perpendicular to its fins fared far worse in comparison to the heat sink made with the melt flow parallel to the fins in both positions.

From these results, it is possible to see that the change of the heat sink’s position does not have an influence if the melt flow is perpendicular to the heat sink’s fins, which means to the direction of gravity and buoyancy. Because of the perpendicularity, the final orientation of additives exhibits a top-to-bottom symmetry in reference to the scheme in 7. Therefore, there is no observed change in cooling efficiency between the positions. 

In the case of the melt flow parallel to the fins, the heat sink’s position plays an important role, even though no visible change is observed on the outside. On the inside, however, the filler orientation changes when reaching the fins’ ends. This change of orientation has a different position to the gravity and air flow induced via natural convection, so the overall heat transfer is different.

**Figure 8:**
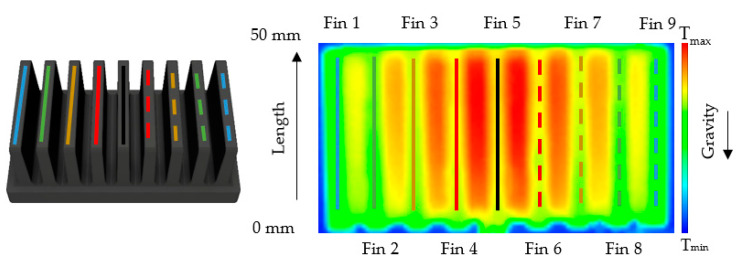
Labelling system of the heat sink’s fins, (**left**)—model of the heat sink, (**right**)—thermal image.

Further analysis is based on the temperature field captured by a thermographic camera. In order to increase fidelity, the data were averaged using several repeated measurements. The labeling scheme of fins is shown in Figure 8. The same colors represent fins of the same distance from the middle of the heat sink. The length of the fins is measured from bottom up, in an opposing direction to gravity.

**Figure 9:**
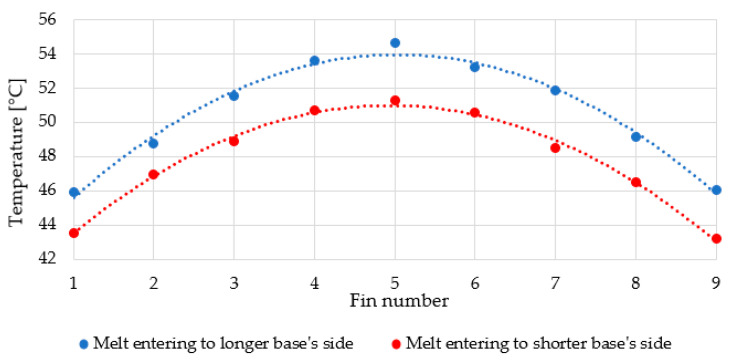
Averaged temperature at the top of fins (fins’ surface furthest from the base) of both measured heat sinks in the “upside-down” position. Each set of temperatures is fitted with a 2nd order polynomial curve.

Comparison of the average fin temperatures is shown in Figure 9. The chart compares the “upside-down” position of the heat sink made with the melt flow parallel to the fins, with the heat sink made with the melt flow perpendicular to the fins.

It is clear that the second heat sink has far cooler fins (individual fin differences range from 1.8 °C to 3.4 °C), which means that the filler does not conduct heat in a desired direction, that is, to the fins’ tops. Therefore, the produced heat is not well distributed in the heat sink’s body, and hence, the heater is warmer.

**Figure 10:**
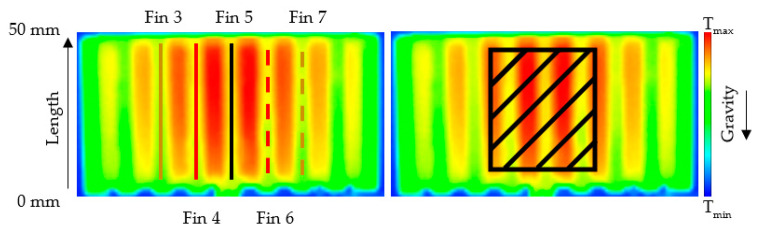
Heater placement in regard to the fins’ positions.

**Figure 11:**
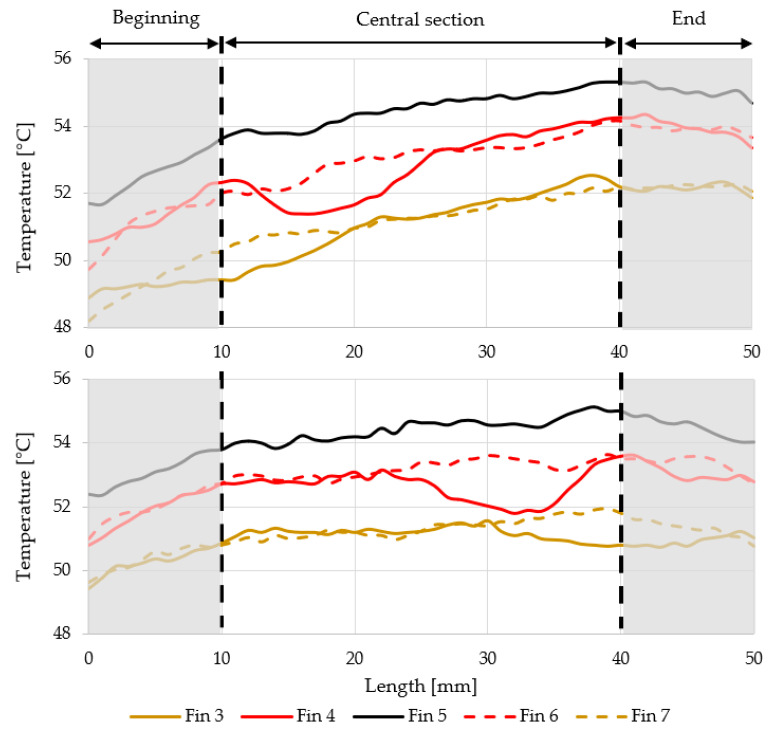
Temperature fields on the heat sink’s fins, (**top**)—“default” position, (**bottom**)—“upside-down” position.

To better understand and see why the position results in different heater temperatures in the case of the heat sink produced with the melt entrance to the longer base’s side, the temperature change along the chosen fins length was investigated. Only the fins closest to the heater are of concern as they are the warmest and have a better potential to conduct heat away from the heater. In Figure 10, the position of the heater and its effect on the temperature field around the middle fins (hot red area) can be observed, and in Figure 11 the temperature change along the chosen fins is shown.

In the case of the “default” position, the temperature rises in the direction of the air flow induced via natural convection, as expected. However, in the “upside-down” position, the temperature field in the central section of the fins’ surface is more evenly distributed. The fins’ conductivity is not symmetrical with respect to its center (length = 25 mm). By turning the heat sink to the “upside-down” position, the more conductive part moves down and comes into contact with the flowing air first. As a result, the temperature field on the fins is more homogeneous, and therefore, more heat dissipates into the ambient air temperature.

In the “upside-down” position, the difference between the end and the beginning of the top of the fins is roughly 60% on average, compared to the “default” position. It means that the top of the fins is locally able to transfer more heat to the ambient air temperature.

**Figure 12:**
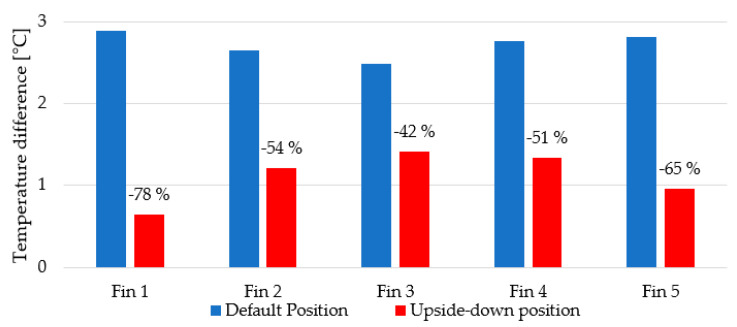
The difference between averaged temperature at the fins’ ends (40–50 mm) and the fins’ beginnings (0–10 mm). The relative drop in percentages of “upside-down” configuration is displayed over red bars.

To quantify the overall difference, the measured temperature at the beginning (0–10 mm) and at the end of the fins (40−50 mm) was averaged. The differences between these averages are shown in Figure 12.

### 4.2. Heat Flow Comparison

Another approach to the efficiency comparison is analysis of the amount of heat leaving the heat sink’s fins. Since these are the parts that are the furthest from the heater, more heat transferred through fins indicates a better cooling efficiency. An analysis based on the images taken by a thermographic camera was conducted.

**Figure 13:**
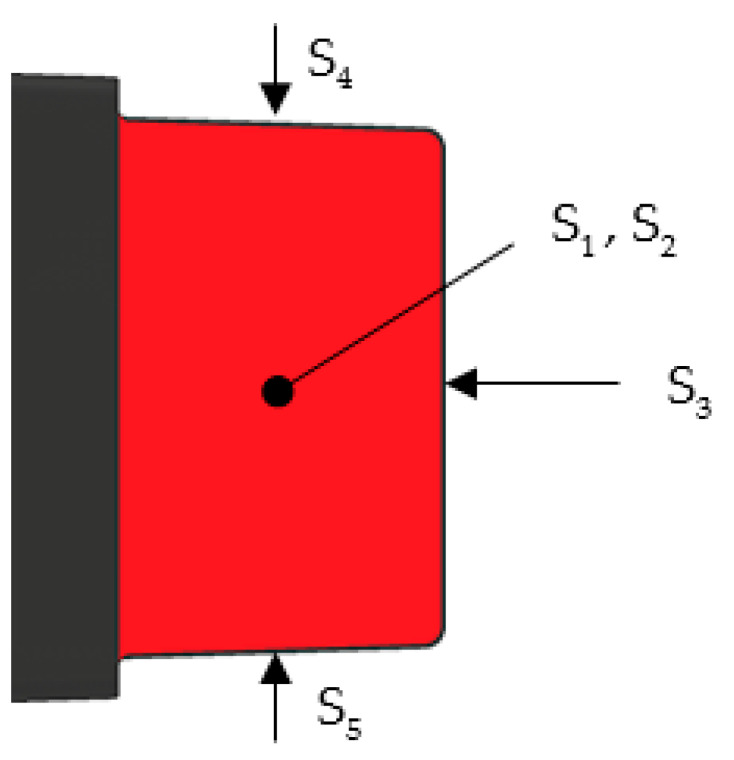
Partition of the fin surface used for the calculations of heat flow.

The calculations were performed according to [16]. The heat flow is composed of convection and radiation. The whole surface of a fin is divided into five parts based on the fin’s sides, shown in Figure 13.

The temperature on each partitioned surface is estimated using thermal imagery. Then, the temperature differences between the surfaces and the ambient temperature, 20 °C, are calculated:

(1) $$\Delta T_{ij}=T_{fin,ij}-T_{ambient}.$$

The index i refers to the fin number and j to the surface $S_{j}$ from Figure 13. The following steps differ for convection and radiation. In the case of convection, the Rayleigh number for the surfaces is

(2) $$Ra_{ij}=\frac{g\cdot \beta_{air}\cdot \Delta T_{ij}\cdot L_{j}^{3}}{\nu_{air}\cdot \alpha_{air}},$$

where g is gravitational acceleration, $\beta_{air}$ is thermal expansion coefficient of the ambient air, $\nu_{air}$ is kinematic viscosity of the ambient air, $\alpha_{air}$ is thermal diffusivity of the ambient air, and $L_{j}$ is characteristic dimension of the surface $S_{j}$ ($L_{1},L_{2},L_{3}$—fin length, $L_{4},L_{5}$—surface area divided by its perimeter). The air properties are evaluated at so called film temperature

(3) $$T_{film,ij}=\frac{T_{fin,ij}+T_{ambient}}{2}.$$

Subsequently, convective heat transfer coefficient is calculated:

(4) $$h_{conv,ij}=f_{j}\left(Ra_{ij},\;L_{j},k_{air}\right),$$

where $k_{air}$ is ambient air’s thermal conductivity at the film temperature.

**Figure 14:**
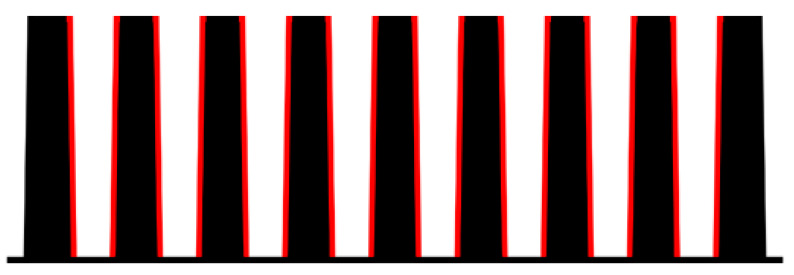
Fins’ surfaces (red) whose radiative heat transfer coefficient needs to be adjusted due to their mutual radiation.

In case of radiation, the radiative heat transfer coefficient is as follows:

(5) $$h_{rad,ij}=\varepsilon \sigma \left(T_{fin,ij}+\;T_{ambient}\right)\cdot \left(T_{fin,ij}^{2}+\;T_{ambient}^{2}\right),$$

where the surface emissivity ε is set to 0.95 and σ is the Stefan–Boltzmann constant. A special treatment is applied to the surfaces whose mutual radiative interaction cannot be neglected (see Figure 14).

Therefore, the ambient view factor, $F_{amb}$, is calculated for these surfaces. The factor represents the fraction of ambient which is visible from the surface. Its simplified evaluation leads to the form

(6) $$F_{amb}=\frac{A_{amb,1}\;+\;2\cdot A_{amb,2}}{\;A_{gap}\;+\;\;2\cdot A_{fin}}.$$

**Figure 15:**
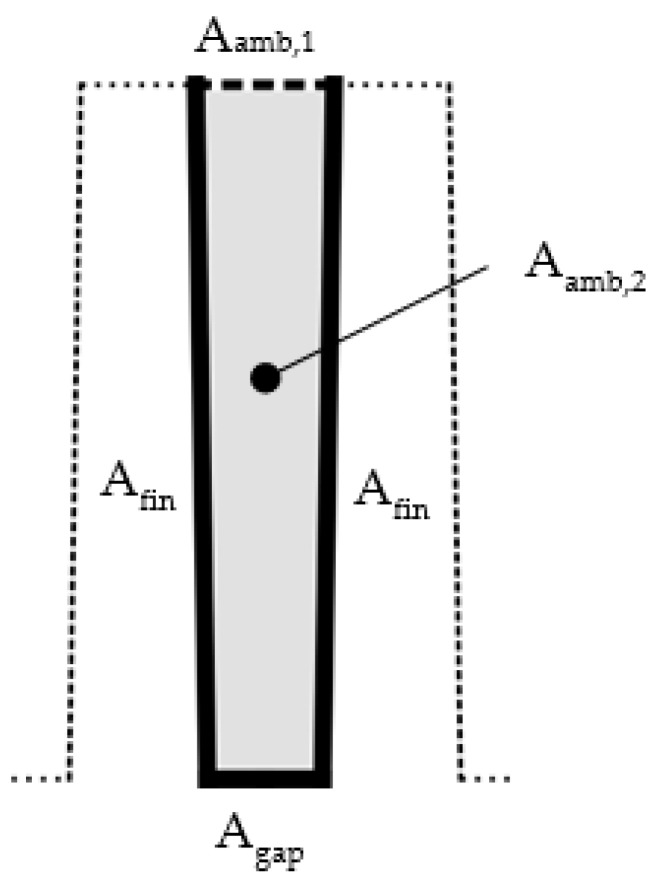
Two-dimensional cut of a simplified fin model used to evaluate the ambient view factor.

The surface areas $A_{amb,1},\;A_{amb,2},\;A_{fin},\;A_{gap}$ are taken from a fin geometry shown in Figure 15**.**

Then, the radiative heat transfer coefficient is modified:

(7) $$h_{rad,ij}^{*}=\;F_{amb}\cdot h_{rad,ij}.$$

For surfaces not affected by mutual radiation, $F_{amb}$ is equal to 1. Finally, the individual heat flows $Q_{i}$ from the fins are

(8) $$Q_{i}=\underset{j}{\sum }Q_{ij},\;\;\;\;\;\;\;Q_{ij}=\left(h_{conv,ij}+h_{rad,ij}^{*}\right)\cdot A_{j}\cdot \Delta T_{ij},$$

where $A_{j}$ is the area of surface $S_{j}$.

**Figure 16:**
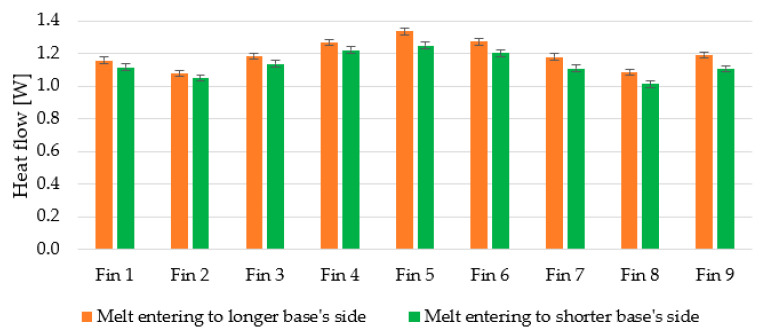
Comparison of individual heat flows leaving fins of heat sinks in the “upside-down” position differing in the melt entry.

The heat flows through individual fins are displayed in Figure 16, which compares heat sinks placed in the “upside-down” position. It is clear to see that the amount of transferred heat via fins is higher in the case of the melt entering through the long side of the heat sink’s base. Moreover, the influence of radiation on the outermost fins is evident as more of their surface area is open to the ambient air temperature. The error bars represent the thermography measurement error of ±0.5 °C.

**Figure 17:**
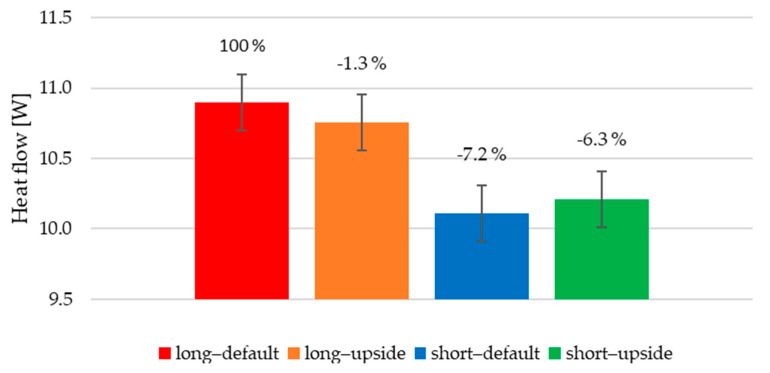
Total calculated heat flow leaving fins for all configurations of conducted measurements.

To get the overall heat flow leaving from all the fins, the individual flows were summed. These values range between 10.1 and 10.9 W. To get a better view of the difference, the flows are related to the sink with the melt flowing to the longer base’s side, configured in the “default” position. The percentage drops are displayed in Figure 17, over the corresponding bars. The error bars represent the thermography measurement error of ±0.5 °C.

As far as different positions of the same heat sink are concerned, the difference of up to 2% is negligible and can be explained by the measurement error of the thermal imagery and by the assumptions on the calculation procedure. One of those is the isothermal surface, which in our case was not met (the average difference between maximal and minimal measured temperature on the fins’ tops across all measurements is 3.5 °C). Furthermore, heat transfer from the heat sink base is not included in the calculations. Comparing the heat sinks with different melt flow, a 6–7% heat flow drop can be observed. This leads to the conclusion that heat sinks made with the entry leading to the longer side of the base and therefore parallel to the fins transfer heat more efficiently.

## 5. Conclusions

Thermally conductive polymers are a new type of composite material that expands the potential applications of polymers. During its mold design, it is important to focus not only on the resulting surface quality and mechanical properties as usual, but also on the orientation of additives in the final product. The orientation of the additives in the product can be suitably influenced by the choice of the mold geometry, the parameters of the injection, and the injection system.

The importance of the melt flow gate for production of polymer heat sinks was shown. Choosing the correct melt entrance into the mold’s cavity can significantly improve the heat sink’s cooling efficiency, which means that the correct design of melt flow during the production is important.

This article also shows the dependency of the orientation of the highly thermally conductive filler in regard to buoyancy, while in use on the overall cooling efficiency. While the heat sink produced with melt flowing perpendicularly to the buoyancy orientation did not show any cooling efficiency changes in the two measured positions, the heat sink produced with the melt flowing parallelly to the buoyancy orientation showed different cooling efficiency.

In electronics cooling applications, the most important indicator of cooling efficiency is the integrated circuit’s temperature. For this purpose, the individual heat sinks and their positions are compared based on the total heating of the heater (see Table 2), which was calculated as the difference between the heater and the ambient air temperature, maintained at 20 °C. Measured heat sinks were subjected to the thermal power input of 15 W. Concerning the same geometry, changing the melt entry during production and spatial orientation during the use of the heat sinks led to an almost 13% improvement in cooling efficiency (the temperature of the heater dropped from 76.2 °C to 69.5 °C).

## Figures and Tables

**Table 1:** Temperatures on the heater in all types of conducted measurements.

	Default Position [°C]	Upside-Down [°C]	Position Difference [°C]
Melt entrance along the longer base’s edge (parallel to fins)	72.3	69.5	2.8
Melt entrance along the shorter base’s edge (perpendicular to fins)	76.1	76.2	0.1
Melt Entry Difference [°C]	3.8	6.7	

**Table 2:** Relative comparison of the heat sinks and their position with respect to the difference between heater and ambient temperature. Heat sinks differed in melt entry point. The first one was made with the melt flowing to the longer side of its base (LONG), parallel to the fins and buoyancy; the second one had the entry leading to the shorter side (SHORT), perpendicular to the fins and buoyancy. Both were measured in two space configurations—a reference position (DEFAULT) and turning the heat sink 180° around its shorter side (UPSIDE-DOWN).

LONG—DEFAULT	LONG—UPSIDE-DOWN	Difference
100%	94.9%	5.1%
SHORT**—**UPSIDE-DOWN	LONG**—**UPSIDE-DOWN	
100%	87.4%	12.6%
SHORT**—**DEFAULT	LONG**—**DEFAULT	
100%	92.5%	7.5%

## Data Availability

Not applicable.
